# Prevalence of Sensitization to Aeroallergens in Greater Beijing Region Children With Respiratory Allergy

**DOI:** 10.3389/fped.2022.848357

**Published:** 2022-05-19

**Authors:** Kai Guan, Wenjing Zhu, Li Sha, Chuanhe Liu, Jing Zhao, Jia Yin, Yuzhi Chen

**Affiliations:** ^1^Department of Allergy, Peking Union Medical College Hospital, Peking Union Medical College, Chinese Academy of Medical Sciences, Beijing, China; ^2^Peking Union Medical College, Beijing Key Laboratory of Precision Medicine for Diagnosis and Treatment of Allergic Disease, Beijing, China; ^3^National Clinical Research Center for Dermatologic and Immunologic Diseases (NCRC-DID), Beijing, China; ^4^Department of Allergy, Children's Hospital of Capital Institute of Pediatrics, Beijing, China

**Keywords:** aeroallergens, respiratory allergy, atopy, skin prick test, children

## Abstract

**Objective:**

To evaluate the prevalence and distribution of sensitization to aeroallergens in children with atopic diseases.

**Methods:**

We conducted skin prick test on 9,527 pediatric patients (aged 0–17 years) with atopic diseases in allergy department of Children's Hospital affiliated with the Capital Institute of Pediatrics. Positive rates of aeroallergens were compared among the different groups.

**Results:**

Boys (69.5%) had a higher positive rate in SPT results than girls (59.8%; χ^2^ = 91.7, *P* < 0.01), and the prevalence of sensitization to aeroallergens increased from 56.1% in the 0–5 year group, to 73.0% in patients above 12 years. Japanese hop (36.2%) and *D. farinae* (28.1%) were the most common outdoor and indoor aeroallergens, respectively. From low to high age groups, the positive detection rates of *D. farinae* were 20.3, 30.6, and 33.5%, respectively, followed by D. pteronyssinus (15.7, 24.8, and 30.0%) and cat dander (8.6, 19.8, and 27.9%, respectively), while dog dander and cockroach showed the same trend. The top three positive detection rates of outdoor aeroallergens were Alternaria (30.9%), Japanese Hop (26.7%), and Artemisia (23.7%) in the preschool age group. With regard to patients aged 6 to 11 years, the three most common were Japanese hop (39.2%), Alternaria (36.4%), and Fraxinus pollen (34.4%). Japanese hop (43.8%), Sabina (41.1%), and birch pollen (39.6%) became increasingly common allergens among adolescents. There were more patients with strongly positive reactions to Alternaria in AS (χ^2^ = 10.2, *P* < 0.01) and AS with AR groups (χ^2^ =9.7, *P* < 0.01) than those in the AR group. Asthmatic patients had significantly higher multiple positive reactions than those with AR (*P* < 0.01). Asthmatic patients had a much higher prevalence of HDM, animal dander, and Alternaria than those with allergic rhinitis (*P* < 0.05).

**Conclusion:**

The prevalence of sensitization to aeroallergens increased with age in children with atopic diseases in Greater Beijing Region. Alternaria was the predominant allergen before 5 years of age, and tree pollen had delayed sensitization in adolescents. Sensitization to perennial allergens such as HDM, cats, and Alternaria was more strongly associated with asthma risk. Sensitization to more than one allergen significantly affected asthmatic patients.

## Introduction

Allergy is on the rise worldwide, affecting approximately 25% of the world's population. Due to its long-term chronic recurrence, it has become a serious problem threatening social and public health (1). Sensitization to aeroallergens is an important pathogenic and risk factor in the development of airway allergic diseases in children (2, 3). Geographical and seasonal variations in aeroallergen sensitization have been observed in China and sensitization patterns also change significantly over time (4). It is essential to identify allergens for diagnosis, allergen-specific immunotherapy, and prevention of these diseases. To fill the knowledge gap due to the lack of recent large sample size studies of aeroallergen sensitization of respiratory allergy in children, we described variation in the prevalence of positive skin prick test (SPT) to common aeroallergens among children under 18 years of age.

## Methods

### Study Design and Population

We conducted a retrospective analysis of patients with atopic diseases who visited the Allergy Department of Children's Hospital affiliated with the Capital Institute of Pediatrics within a 2-year period (2018–2020). The patients were mainly from Greater Beijing region (Beijing, Tianjin and Hebei Province). Allergic rhinitis and asthma were diagnosed by allergists *via* history taking, clinical examination, and spirometry, in accordance to Allergic Rhinitis and its Impact on Asthma (ARIA) and the Global Initiative for Asthma (GINA) guidelines. This study was approved by the ethical committee of our hospital (SHERLL2021011).

### Data Collection

Within the designated time period of 24 months, patient charts were used as the primary source of information regarding outpatient visits in Allergy Department of Children's Hospital of Capital Institute of Pediatrics. The collected data included age, sex, clinical history, SPT results and diagnosis of diseases. After excluding missing data, a total of 9,527 patients aged 0–17 years were enrolled in this study. The children were divided into three groups according to age: preschool (0–5 years), early school age (6–11 years) and adolescents (12–17 years). Those who had a clear diagnosis were classified into different disease groups: allergic rhinitis (AR), asthma (AS) and asthma with allergic rhinitis (AS with AR).

### Skin Prick Test (SPT)

Allergen extracts (produced by the Allergen Products Research and Development Center, Peking Union Medical College's Hospital, Chinese Academy of Medical Sciences, Beijing, China) of *Dermatophagoides farinae, Dermatophagoides pteronyssinus*, cockroach, cat dander, dog dander, tree pollen, weed pollen and molds used to perform allergy test on all patients, and the procedure followed a practical guide (5). Histamine (10 mg/ml) and physiological saline solution were used as positive and negative controls, respectively. Drugs which may have an impact on SPT were withdrawn before the test. Skin response was measured 15 min after pricking. Positive skin prick test was defined as a wheal diameter greater than 3 mm in the presence of a negative saline control. SPT reactivity was graded as the allergen histamine wheal ratio (AHWR) (6). If the grade was +++ or ++++, it was considered a strongly positive reaction. Multiple sensitizations were defined as positive reactions to two or more allergens, including house dust mite (HDM), animal dander, molds, tree pollen and weed pollen.

### Statistics

All data were analyzed using SPSS (SPSS Statistics version 23.0, IBM). Descriptive analyses (mean, standard deviation, range, median, and percentages) were performed. The Mann–Whitney test was used to compare groups that failed to meet the normal distribution. A Chi-squared test was used to qualitatively analyze categorical independent variables. Statistical significance was set at *P* < 0.05.

## Results

### Participant Characteristics

A total of 9,527 patients aged 0–17 years (mean age 7.5 ± 5.8 years) completed the SPT examination and entered the study. Overall, 6,071 (63.7%) were boys (mean age 7.5± 2.5 years) and 3,456 (36.3%) were girls (mean age 7.4 ± 2.7years). A total of 5,100 patients (53.5%) had AR, 1,771 patients (18.6%) had AS, 1,864 patients (19.6%) had AS with AR, and 792 (8.3%) were diagnosed with non-respiratory allergic diseases. Boys (69.5%, 4,218/6,071) had a higher positive rate in SPT results than girls (59.8%, 2,067/3,456; χ^2^ = 91.7, *P* < 0.01). Demographic data are reported in Table 1.

**Table 1:** Demographic characteristics of cases with received SPT.

	**Cases (number)**
**Age (years)**	
0–5	2570
6–11	6,178
12–17	779
**Gender**	
Female	6,071
Male	3,456
**Disease**	
Allergic rhinitis	5.100
Asthma	1.771
Asthma with allergic rhinitis	1.864
Non-respiratory allergy	792

### Prevalence of Sensitization to Aeroallergens in Different Groups

**Figure 1:**
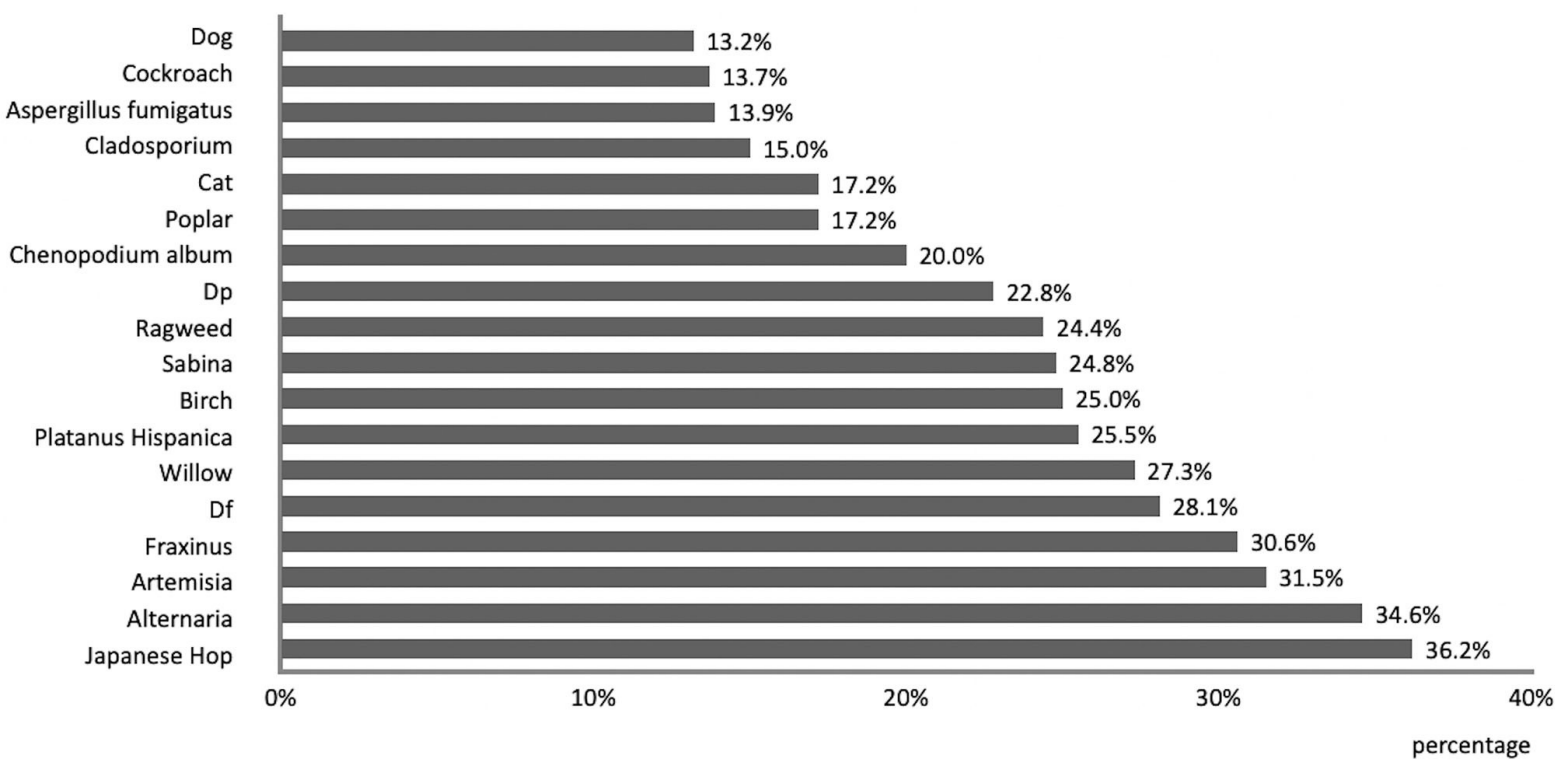
Prevalence of different kinds of aeroallergens.

The top five aeroallergens in terms of positive rates were *Japanese Hop* (36.2%), *Alternaria* (34.6%), *Artemisia* (31.5%), *Fraxinus* (30.64%) and *D. farina* (28.1%; Figure 1).

The prevalence of sensitization to aeroallergens was 56.1% (1,442/2,570) in the 0–5 year-old group, 69.2% (4,274/6,178) in the 6–11 year-old group, and 73.0% (569/779) in the 12–17 year-old group. Significant differences were found among the three groups (*P* < 0.01).

The prevalence of sensitization to aeroallergens was 64.6% (3,297/5,100) in the AR group, 69.6% (1,233/1,771) in the AS group and 70.1% (1,307/1,864) in the AS with AR group, respectively. There were no significant differences between AS and AS with AR groups (*P* > 0.05), but patients in these two groups had significantly higher positive reactions than those with AR (*P* < 0.01).

### Prevalence of Sensitization to Indoor Aeroallergens

**Figure 2:**
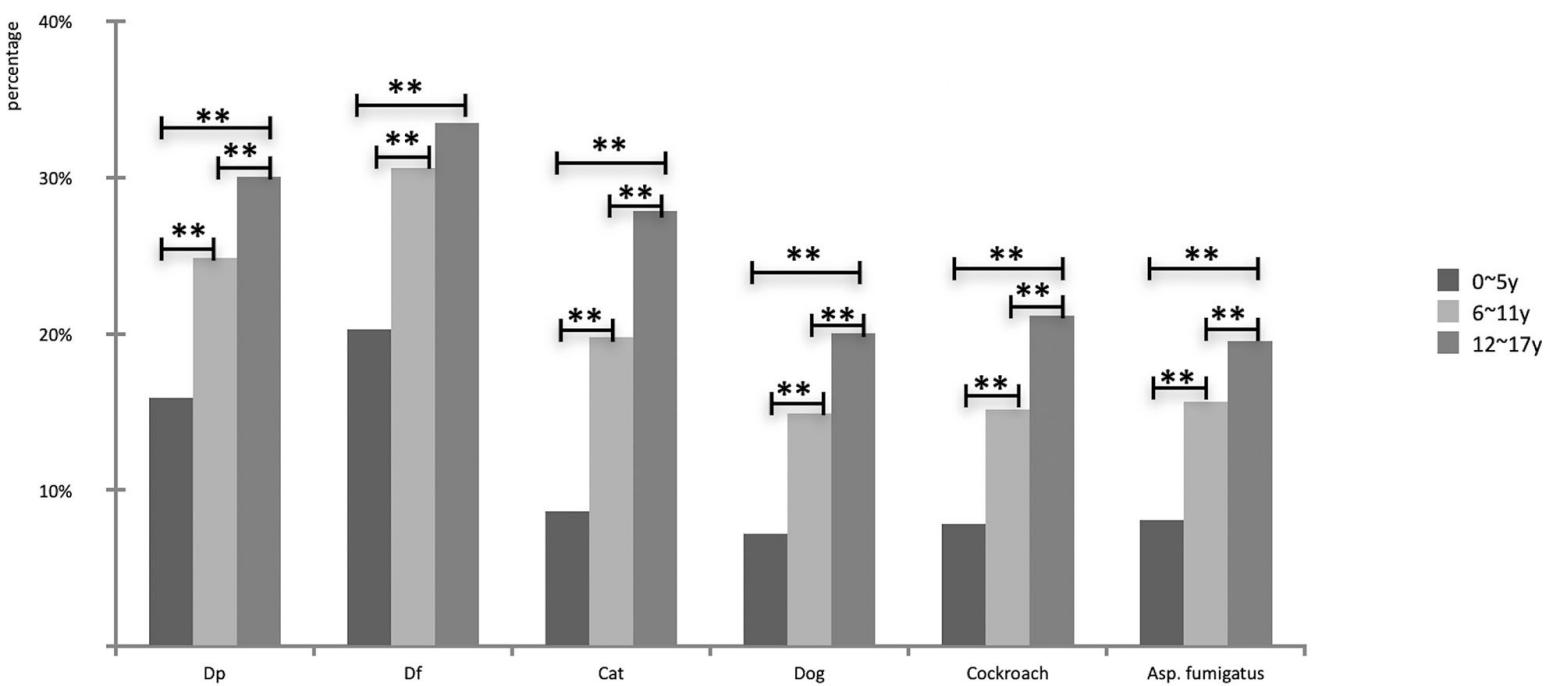
Comparison of prevalence of indoor aeroallergens in different age groups (**P < 0.01).

From low to high age groups, the positive detection rates of *D. farinae* were 20.3, 30.6 and 33.5%, respectively, followed by *D. pteronyssinus* (15.7, 24.8 and 30.0%) and *cat dander* (8.6, 19.8, and 27.9%, respectively), while dog dander and cockroach showed the same trend. Almost all of the groups showed significant differences, except for *D. farinae* between 6–11 and 12–17 years groups (χ^2^ = 2.7, *P* > 0.05; Figure 2).

**Figure 3:**
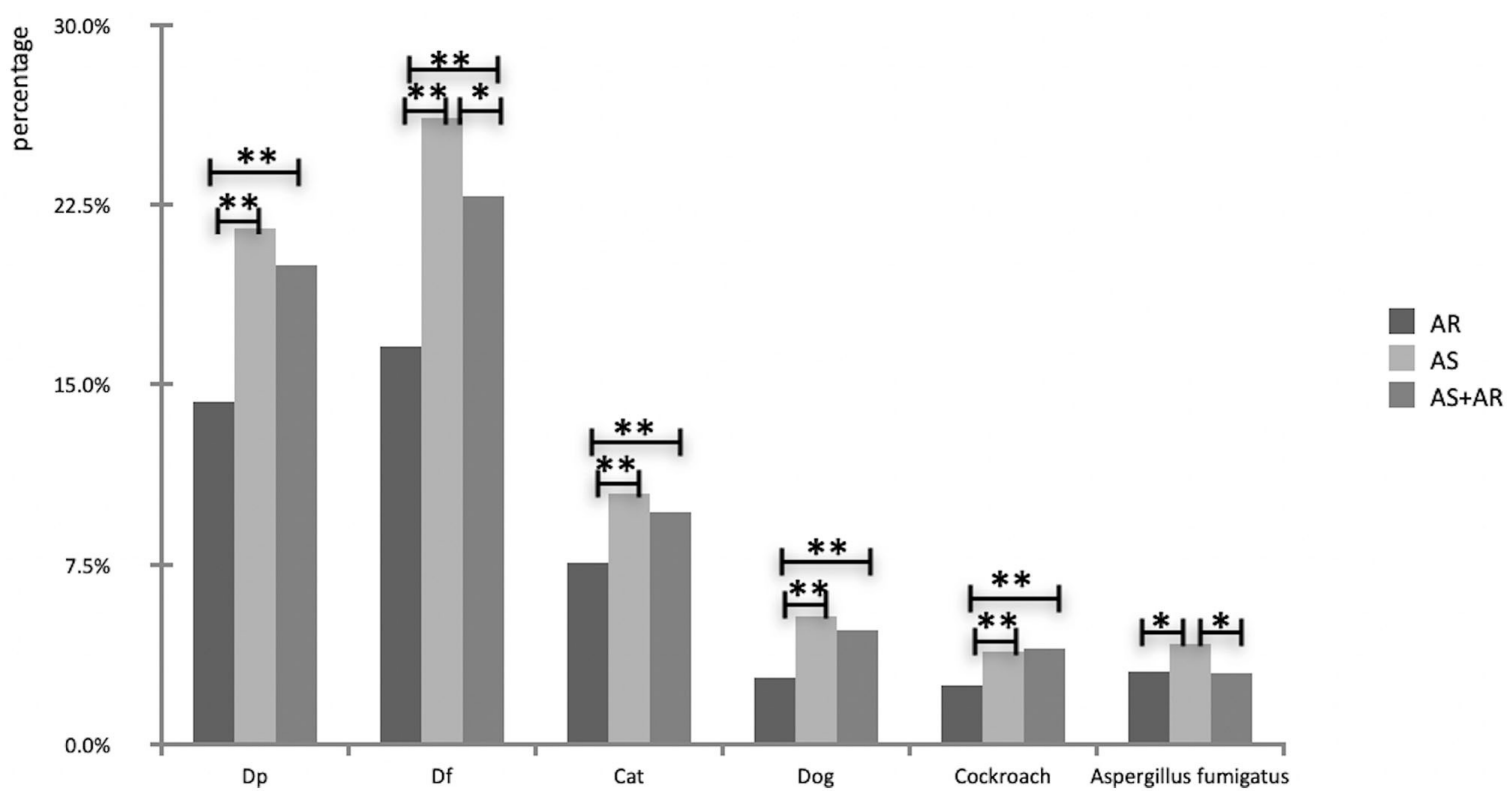
Comparison of prevalence of indoor aeroallergens in different disease groups (*P < 0.05, **P < 0.01).

In different disease groups, the strongly positive detection rates of D. *farinae* were 16.6% in the AR group, 26.1% in the asthma group and 22.9% in the AS with AR group, followed by *D. pteronyssinu*s (14.3, 21.5, and 20.0%) and cat dander (7.6, 10.5, and 9.7%, respectively). The strongly positive detection rates of HDM and cat dander in asthmatic children and patients with AS and AR were significantly higher than those with AR (*P* < 0.05; Figure 3).

### Prevalence of Sensitization to Outdoor Aeroallergens

**Figure 4:**
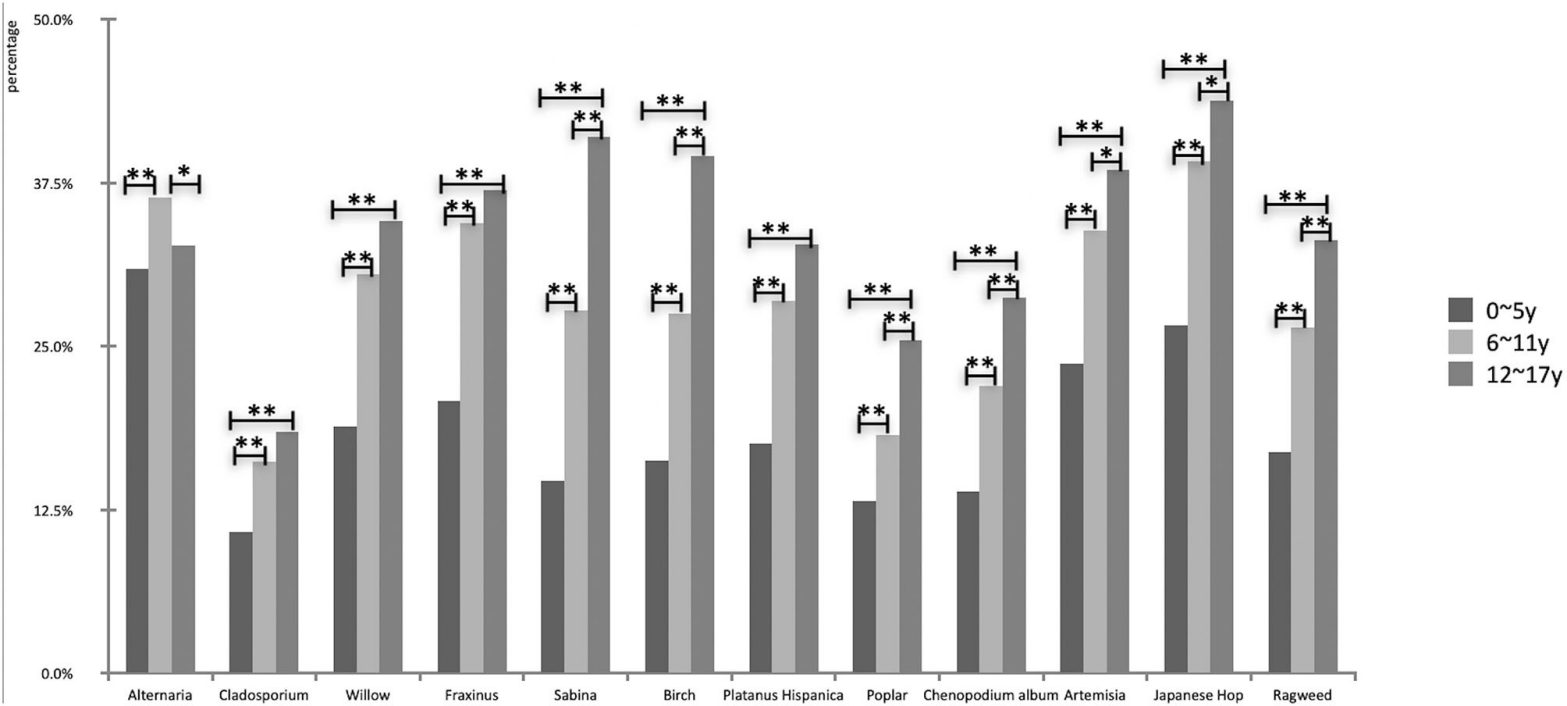
Comparison of prevalence of outdoor aeroallergens in different age groups (*P < 0.05, **P < 0.01).

The prevalence of sensitization to outdoor allergens varied greatly among the different age groups. The top three allergens with regards to positive detection rates of outdoor aeroallergens were *Alternaria* (30.9%), *Japanese Hop* (26.7%) and *Artemisia* (23.7%) in the preschool age group. With regards to patients aged 6–11 years, the first three were *Japanese Hop* (39.2%), *Alternaria* (36.4%), and *Fraxinus pollen* (34.4%). Meanwhile, *Japanese Hop* (43.8%), *Sabina* (41.1%), and *birch pollen* (39.6%) became increasingly common among adolescents (Figure 4).

**Figure 5:**
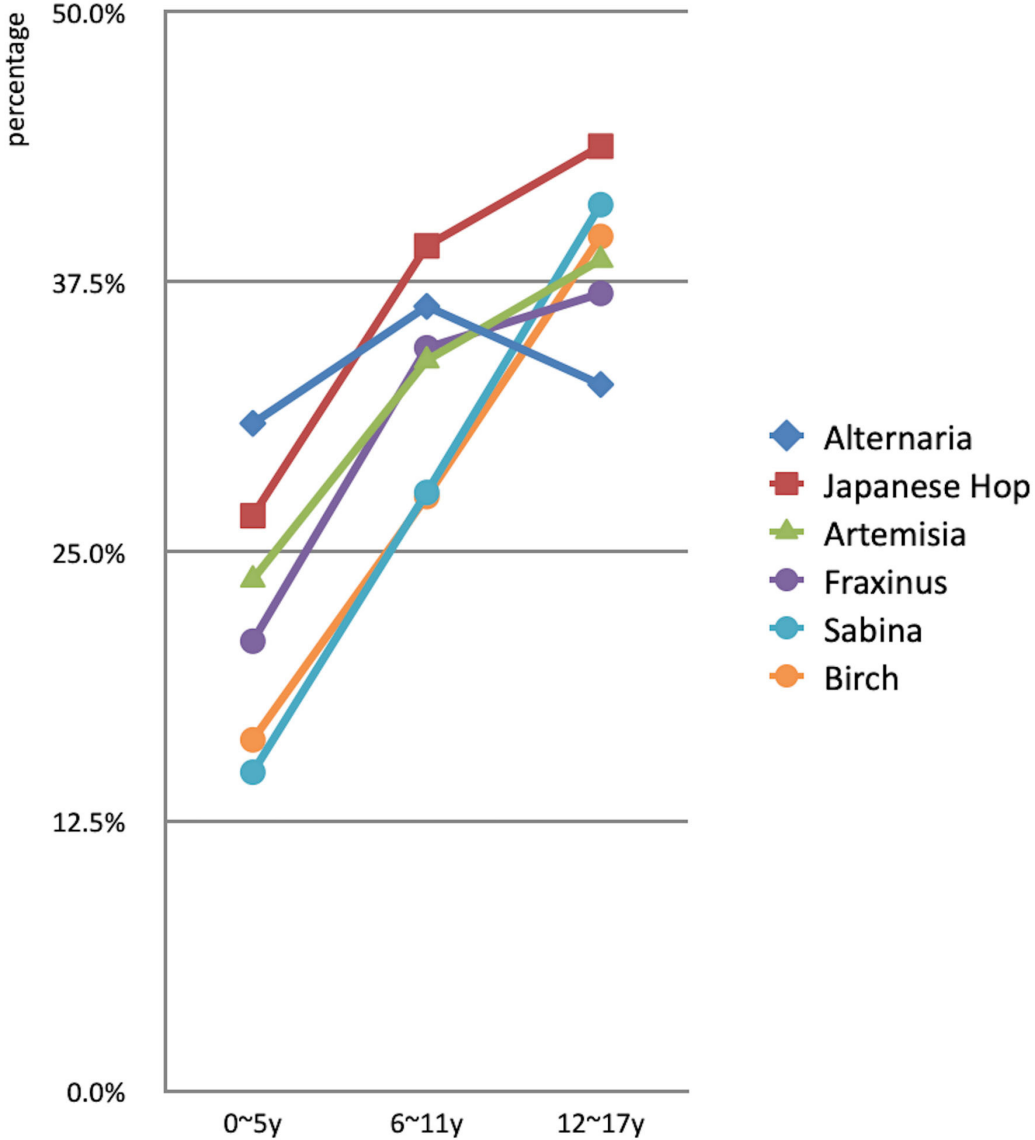
Trend chart of strongly prevalence of outdoor aeroallergens in different age groups.

The prevalence of aeroallergens showed that the proportional increase was consistent with age, except for *Alternaria* (*P* < 0.01; Figure 3). *Alternaria* had the highest positive rate in early school age before 12 years (χ^2^ = 23.5, *P* < 0.01, compared to 0–5-year; χ^2^ = 3.9, *P* < 0.05, compared to the 12–17-year group). The adolescents had similar results to the early school age group, and they were both significantly higher than those of preschool patients (*P* < 0.01). In preschool and school-age patients, the important airborne pollens were mainly composed of weed pollen, including *Japanese Hop* and *Artemisia*, while in adolescents these were *Japanese Hop* and tree pollen (*Sabina* and *Birch*; Figure 5).

**Figure 6:**
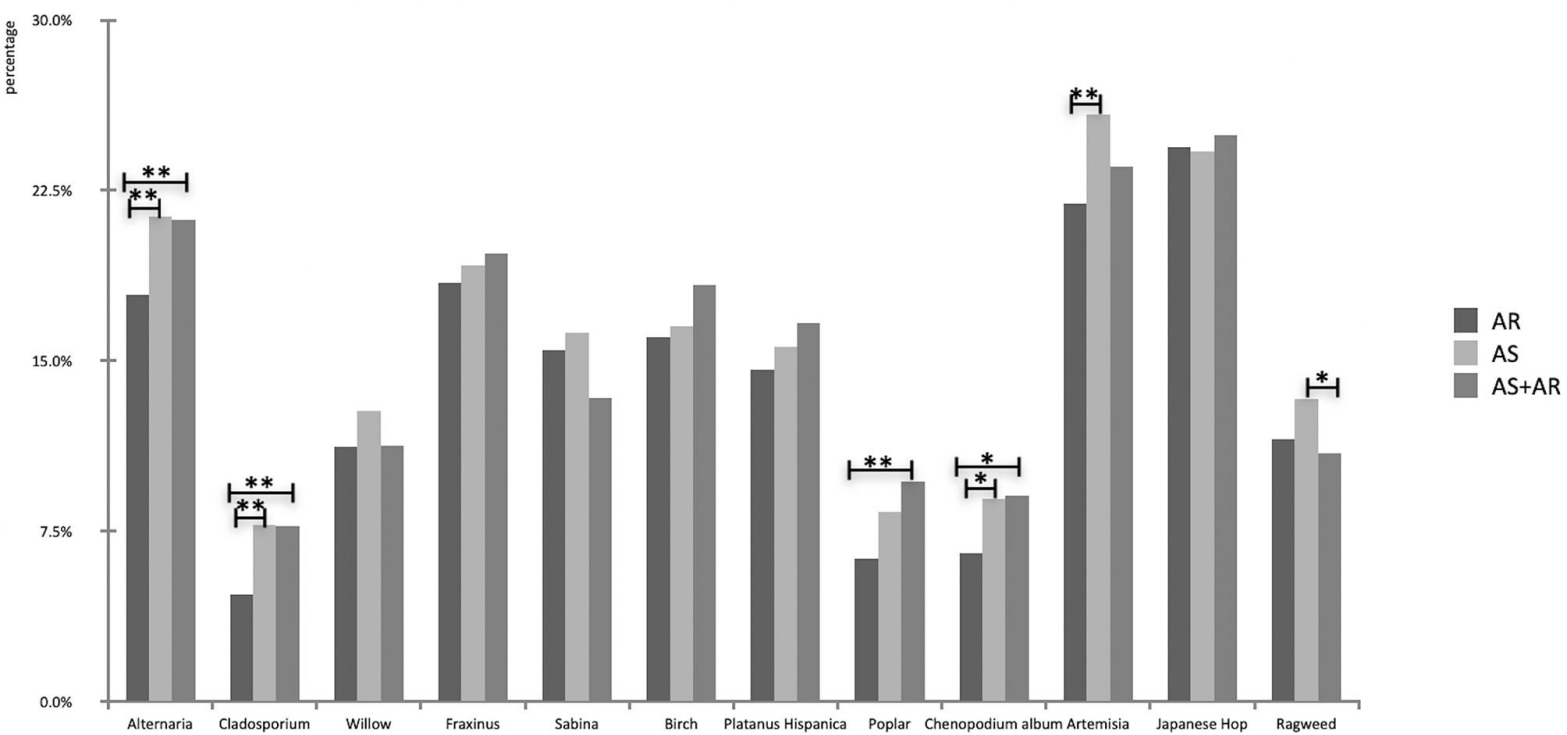
Comparison of prevalence of outdoor aeroallergens in different disease groups (*P < 0.05, **P < 0.01).

In the AR group, the top three strongly positive sensitization to aeroallergens were pollens: *Japanese Hop* (24.2%), *Artemisia* (21.9%), and *Fraxinus* (18.4%). Weed pollen, including *Artemisia, Japanese Hop*, and *Alternaria*, were the main sensitization allergens in patients with AS (25.86, 24.22, and 21.34%, respectively), as well as the AS with AR group (25.0, 23.6, and 21.2%, respectively; Figure 6).

There were more patients with strongly positive reactions to *Alternaria* in AS (χ^2^ = 10.2, *P* < 0.01) and AS with AR groups (χ^2^ = 9.7, *P* < 0.01) than those in the AR group. Nearly no statistically significant differences were found in strongly positive sensitization to pollen among the three groups (*P* > 0.05), except that patients with rhinitis had a lower proportion of sensitization to *Poplar, Chenopodium album*, and *Artemisia* pollen than asthmatic children (χ^2^ = 11.5, *P* < 0.05; Figure 6).

### Prevalence of Multiple Sensitizations to Aeroallergens

**Figure 7:**
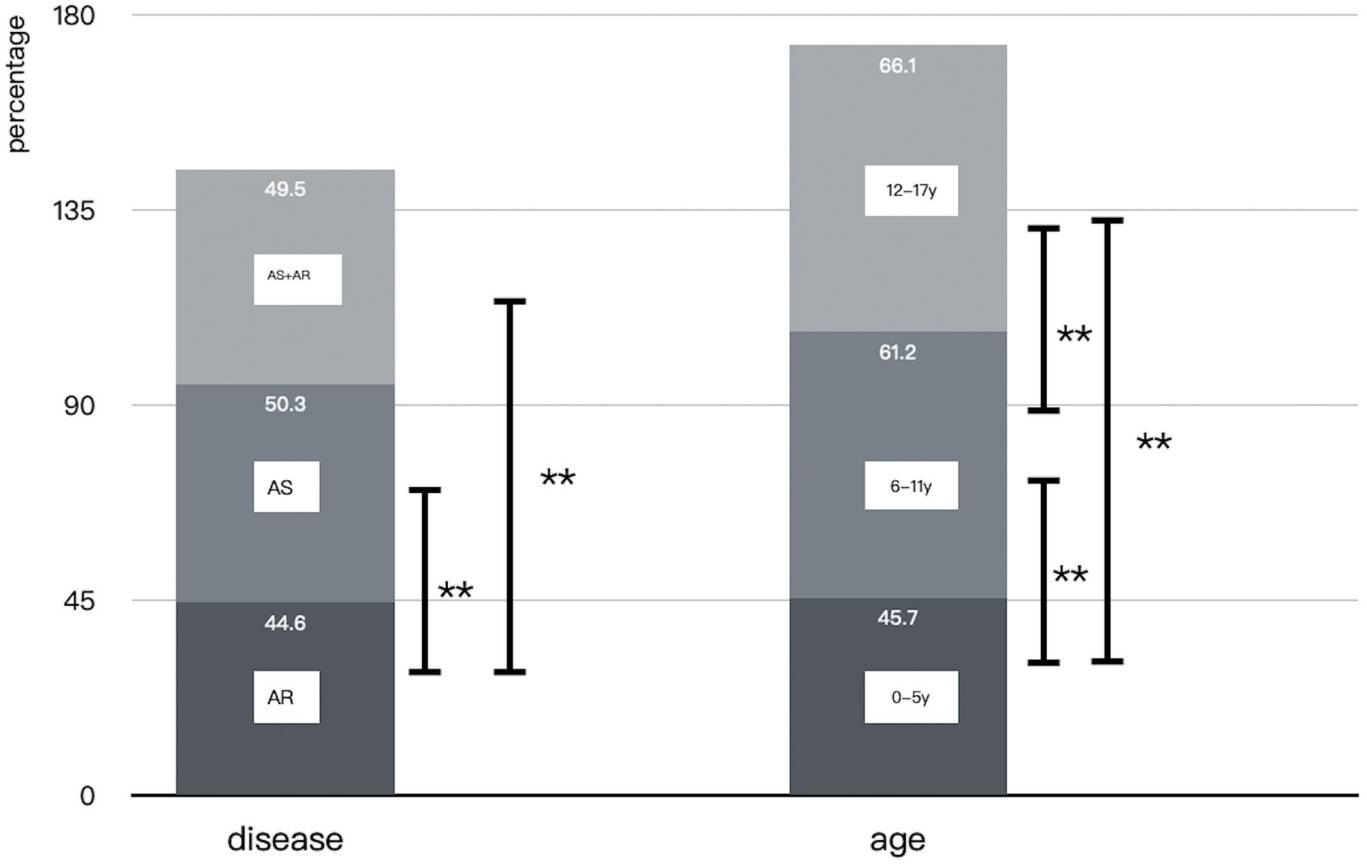
Comparison of multiple sensitizations in different groups (**P < 0.01).

Multiple sensitizations with aeroallergens were 45.7% (1,175/2,570) in the preschool age group, 61.2% (3,781/6,178) in 6–11 years, and 66.1% (515/779) in adolescents, respectively. Statistical differences were found among the groups (*P* < 0.01; Figure 7).

The prevalence of multiple sensitizations was 44.6% (2,273/5,100) in the AR group, 50.3% (890/1,771) in the AS group, and 49.5% (922/1,864) in the AS with AR group. No significant difference was found between asthmatic children with and without allergic rhinitis (*P* > 0.05), but asthmatic patients had significantly higher multiple positive reactions than those with AR (*P* < 0.01; Figure 7).

**Figure 8:**
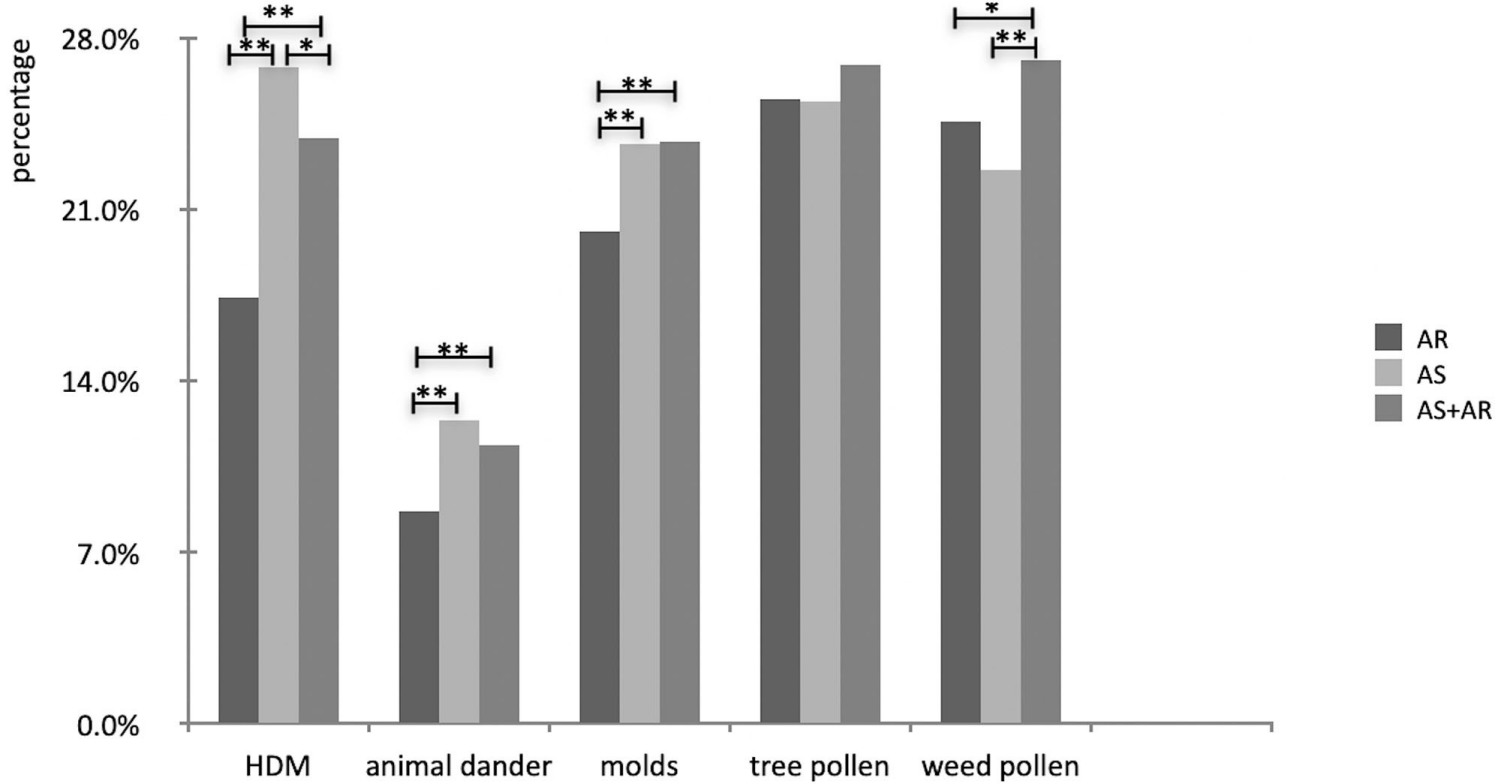
Comparison of prevalence of aeroallergens in different disease groups (*P < 0.05, **P <0.01).

Asthmatic patients had a much higher prevalence of *HDM, animal dander* and *Alternaria* than those with allergic rhinitis (*P* < 0.05). No differences were found in the prevalence of tree pollen while AS with AR group had a higher prevalence of weed pollen than the other two groups (*P* < 0.05); (Figure 8).

## Discussion

Atopic diseases, including allergic rhinitis, asthma, atopic dermatitis and food allergy, are considered epidemic diseases in the 21st century, with increasing prevalence worldwide. The prevalence of allergic rhinitis, asthma, and atopic dermatitis in children has risen significantly in China (7–9). These diseases impose heavy medical burden on public health and families worldwide. Allergen sensitization is an important risk factor for the development, persistence, and severity of atopic diseases. At the early stage of allergic march, the identification of sensitization to specific allergens in children is important for environmental interventions and medical treatment. This study retrospectively analyzed the SPT results of 9527 patients to help clarify allergen profiles in children with respiratory allergies in Greater Beijing Region.

Gender disparity in asthma began early in childhood and influenced the development of asthma with a higher prevalence and more wheezing in boys than in girls. Our previous studies also showed that boys had a much higher prevalence of asthma and food allergies than girls in China (9, 10). Boys had increased atopy compared to girls prior to adolescence, as measured by specific IgE or SPT to common allergens (11–13). The results of this survey suggest that boys with atopic diseases had a higher prevalence of aeroallergens. All these findings indicated that atopy and allergen sensitization were more common in boys than in girls, and may provide a rationale for increased gender disparity in asthma and other atopic diseases prevalence in children.

Similar to many other Asian countries, HDM was the most predominant sensitized allergen in the indoor environment in this study. Among the positive subjects, *D. farinae, D. pteronyssinus* and *cat dander* were the most common indoor inhaled allergens. Differences in aeroallergen sensitization with age were observed. The prevalence of sensitization to *D. farinae* was approximately 20.3% in children under 5 years of age, and increased with age to 33.5% in those older than 12 years. Similar trends were observed for *D. pteronyssinus* and cat dander. This pattern of increasing HDM sensitization with age has also been observed in other Asian children with asthma (14, 15). HDM sensitization is closely associated with wheezing and asthma in atopic children. A multicenter study showed that sensitization to aeroallergens was positively associated with asthma and an earlier onset of more severe allergic airway diseases in China (16). Sensitization to HDM was the main risk factor for the increased wheezing in 13–14 years old schoolchildren in Guangzhou (3). We also found the relationship between strongly positive sensitization to HDM and asthma was much closer.

In Western countries, animal dander is the most common aeroallergen in children. Within the past decade, the prevalence of sensitization to pet allergens in patients with AR increased at an annual rate of 1.3% in Guangzhou (17). Sensitization to pet allergens in asthmatic children was as high as 39.25% in this survey, which was much higher than that observed 10 years ago (18, 19). According to relevant reports, by 2019, there were 73.55 million pet owners in cities and towns in China. Cat dander was the second most sensitized indoor allergen in addition to HDM, which may be due to an increasing number of cat lovers. A cohort study showed that animal dander (particularly cat) sensitization during the first 6 years of life was independently associated with increased asthma and rhinitis risk, regardless of the respective exposure at birth (20). The prevalence of strongly positive sensitization to *cat* dander was much higher in asthmatic children regardless of whether they had rhinitis comorbidity. These findings indicate HDM and cats are important indoor allergens associated with an increased asthma risk in atopic children, and HDM and cats should be highly considered in the management of indoor inhaled allergens.

Age distribution seems to play an important role in outdoor aeroallergens. Sensitization rate to tree pollen and weed pollen were both increased with age. The overall prevalence of *Alternaria* sensitization in children was 34.6%, and predominant sensitization were in patients aged 6–11 years, which is in accordance with the prevalence and age trend reported (21–23). An interesting finding was that *Alternaria* was the most common aeroallergens in preschool children, with a peak *Japanese Hop* sensitization in the other two groups. Additionally, the prevalence of sensitization to tree pollen (*Sabina* and *Birch*) was higher that of other species of pollens, and *Sabina* was second only to *Japanese Hop* in adolescents. Thus we concluded that weed pollen allergens were more important in the early stage of life in children with atopic diseases, and the peak age of sensitization to tree pollen was delayed in adolescents. The occurrence and development of pollen allergy mainly depends on the genetic susceptibility and exposed pollen concentration. Unlike the combined weed pollen, which typically has a much longer flowering season with more days of higher pollen concentrations, the individual tree pollen has peak times over short periods of 2–4 weeks with lower concentrations. In addition, tree pollen seasons can overlap significantly, and the release of pollen from some species is known to occur over a short time period, and may only reach high levels for a few days (24). Tree pollen accounted for 30% of the total annual pollen and while weed pollen nearly double that of amount in Beijing, and weed pollen had a stronger sensitized capability (25). We speculated that tree pollen had to take a relatively longer period of repeated exposure to achieve sensitization in childhood because of its shorter flowering time, lower concentration and sensitized capability.

The sensitization rate to pollens was almost universally low among all the Asian countries except for India and Japan, while atopic patients in Europe and the United States were more highly sensitized to pollens (26). Compared to previous studies, which showed atopic children mostly sensitized to HDM in 2001 and more sensitive to molds in 2010 (18, 19), we found pollen had replaced HDM as well as molds as the most predominant aeroallergen in this survey. Japanese hops (36.2%) and Fraxinus (30.64%) were the most common pollens in autumn and spring, respectively. Over the last few decades, climate change due to global warming, carbon dioxide emissions, and changing rainfall patterns have affected the global distribution of aeroallergens and their sensitization modes. The variation in sensitization to airborne pollens was mainly due to species and quantities of pollen had rapidly increased, and pollen abundance in the air of Beijing increased by 2.83 times within 15 years, which led to the incidence rate of pollinosis correspondingly increasing at the same time (27, 28). In addition, some other possible reasons for changes in aeroallergen sensitization patterns included rapid urbanization, air pollution, improved hygiene conditions, and more attention to children's lifestyle, such as increased outdoor activities.

The prevalence of sensitization to aeroallergens as well as multiple sensitizations increased with age, which indicated that increasing age predicted allergic sensitization and multiple allergen sensitizations (29). When children with allergy and symptoms of asthma were studied, the frequencies of HDM, animal dander and molds sensitivity were higher than those with allergic rhinitis alone. Among the perennial allergens, HDM, cats and *Alternaria* were most closely related to asthma. Children are exposed to most indoor aeroallergens in early childhood and as perennial allergens, they have to be continuously exposed throughout the year, so these allergens are more closely related to asthma. Accordingly, pediatricians should advise avoidance of known allergens in children with high risk of respiratory allergy. To those with AR or AS who have identified allergens that correlate with clinical symptoms, environmental controls (i.e, bed covers and acaricides, removal of pets, the use of dehumidifier and air filtration systems, etc) should be recommended. Studied have shown multiple sensitization status at all single time-points associated with an increased risk of asthma at age 13 whereas monosensitization was inconsistently associated with asthma (30). We also found that the asthma group had more patients with multiple sensitizations to aeroallergens than the AR group, making the repetitive sensitization measurements essential when assessing the risk of developing asthma.

### Limitations

This survey was a retrospective study conducted in a children's hospital in Greater Beijing Region. Patients in this study might not be representative. However, it is a large sample population covering almost all ages of children. We carried out a comprehensive and detailed aeroallergen SPT measurement and compared the results with those of our previous studies.

In conclusion, this study revealed that prevalence of sensitization to aeroallergens increased with age in children with atopic diseases in Greater Beijing Region. *Alternaria* was the predominant allergen before 5 years, and tree pollen had a delayed sensitization in adolescents. Sensitization to perennial allergens such as HDM, cats and *Alternaria* was more strongly associated with asthma risk. Sensitization to more than one allergen significantly increased the prevalence of asthma. The identification of specific allergens makes them potential targets for intervention and prevention strategies.

## Data Availability Statement

The raw data supporting the conclusions of this article will be made available by the authors, without undue reservation.

## Ethics Statement

The studies involving human participants were reviewed and approved by the Ethical Committee of the Capital Institute of Pediatrics. Written informed consent from the participants' legal guardian/next of kin was not required to participate in this study in accordance with the national legislation and the institutional requirements.

## Author Contributions

LS and JY designed the study and revised the manuscript. KG drafted the manuscript. WZ analyzed the data and revised the manuscript. LS, CL, JZ, and YC enrolled patients and revised the manuscript. All authors had final approval of the version to be published.

## Funding

CAMS Innovation Fund for Medical Sciences (2020-I2M-C&T-B-007), KG Project Leader; Beijing Hospital Administration Scientific Research Training Program (PX2021051), LS Project Leader. No funds received for open access publication fees from our institution, library, and other grants.

## Conflict of Interest

The authors declare that the research was conducted in the absence of any commercial or financial relationships that could be construed as a potential conflict of interest.

## Publisher's Note

All claims expressed in this article are solely those of the authors and do not necessarily represent those of their affiliated organizations, or those of the publisher, the editors and the reviewers. Any product that may be evaluated in this article, or claim that may be made by its manufacturer, is not guaranteed or endorsed by the publisher.
